# 4-Hexyl­oxybenzamide

**DOI:** 10.1107/S1600536811003096

**Published:** 2011-02-12

**Authors:** Huey Chong Kwong, Mohamad Zaki Ab. Rahman, Mohamed Ibrahim Mohamed Tahir, Sidik Silong

**Affiliations:** a Department of Chemistry, Faculty of Science, Universiti Putra Malaysia, 43400 UPM Serdang, Selangor, Malaysia

## Abstract

In the title compound, C_13_H_19_NO_2_, the dihedral angle between the benzene ring and the plane throught the non-H atoms of the amide group is 29.3 (1)°. The benzene ring and the alkane carbon skeleton plane are twisted slightly with respect to each other [5.40 (5)°]. In the crystal, mol­ecules are oriented with the amide groups head-to-head, forming N—H⋯O hydrogen-bonded dimers. The dimers are connected by further N—H⋯O hydrogen bonds into a ladder-like motif along the *b* axis.

## Related literature

For standard bond lengths, see Allen *et al.* (1987 ▶). For related structures, see: Merz (2002 ▶); Jones *et al.* (2002 ▶); Pagola & Stephens (2009 ▶); Boese *et al.* (1999 ▶). For related experiments on the hydrolysis of nitrites, see: Gallardo & Begnini (1995 ▶); Pala Wilgus *et al.* (1995 ▶).
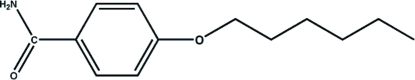


## Experimental

### 

#### Crystal data

C_13_H_19_NO_2_
*M* *_r_* = 221.30
Monoclinic, 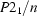

*a* = 12.5507 (2) Å
*b* = 5.16441 (9) Å
*c* = 18.9322 (3) Å
β = 91.4702 (16)°
*V* = 1226.72 (4) Å^3^
*Z* = 4
Cu *K*α radiation
μ = 0.64 mm^−1^
*T* = 150 K
0.31 × 0.08 × 0.05 mm

#### Data collection

Oxford Diffraction Gemini E diffractometer
Absorption correction: multi-scan (*CrysAlis PRO*: Oxford Diffraction, 2006 ▶) *T*_min_ = 0.894, *T*_max_ = 1.000
13258 measured reflections
2355 independent reflections
2165 reflections with *I* > 2σ(*I*)
*R*_int_ = 0.018

#### Refinement

*R*[*F* ^2^ > 2σ(*F* ^2^)] = 0.037
*wR*(*F* ^2^) = 0.095
*S* = 1.01
2101 reflections
145 parameters
H-atom parameters constrained
Δρ_max_ = 0.18 e Å^−3^
Δρ_min_ = −0.18 e Å^−3^

### 

Data collection: *CrysAlis CCD* (Oxford Diffraction, 2006 ▶); cell refinement: *CrysAlis RED* (Oxford Diffraction, 2006 ▶); data reduction: *CrysAlis RED*; program(s) used to solve structure: *SIR92* (Altomare *et al.*, 1994 ▶); program(s) used to refine structure: *CRYSTALS* (Betteridge *et al.*, 2003 ▶); molecular graphics: *Mercury* (Macrae *et al.*, 2006 ▶); software used to prepare material for publication: *CRYSTALS*.

## Supplementary Material

Crystal structure: contains datablocks global, I. DOI: 10.1107/S1600536811003096/kp2300sup1.cif

Structure factors: contains datablocks I. DOI: 10.1107/S1600536811003096/kp2300Isup2.hkl

Additional supplementary materials:  crystallographic information; 3D view; checkCIF report

## Figures and Tables

**Table 1:** Hydrogen-bond geometry (Å, °)

*D*—H⋯*A*	*D*—H	H⋯*A*	*D*⋯*A*	*D*—H⋯*A*
N10—H101⋯O9^i^	0.90	2.14	3.0153 (18)	164
N10—H102⋯O9^ii^	0.90	2.04	2.9401 (18)	174

Symmetry codes: (i) 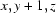; (ii) 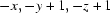.

**Fig. 1.**
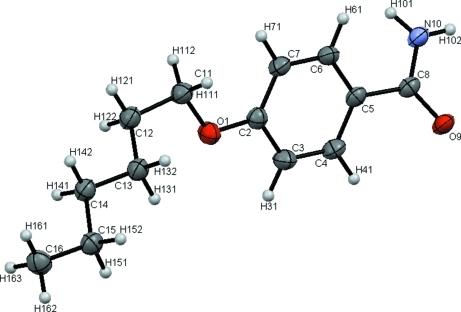
Molecular structure of (I) with arom numbering and displacement ellipsoids at the 50% probablity level.

**Fig. 2.**
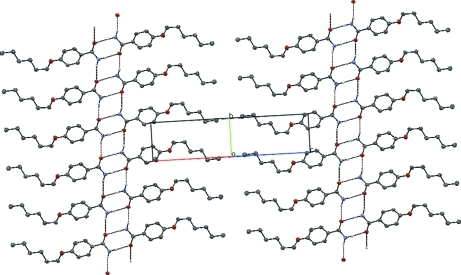
The hydrogen-bonded ladder-like motif (dashed lines) extends along b-axis.

**Fig. 3.**
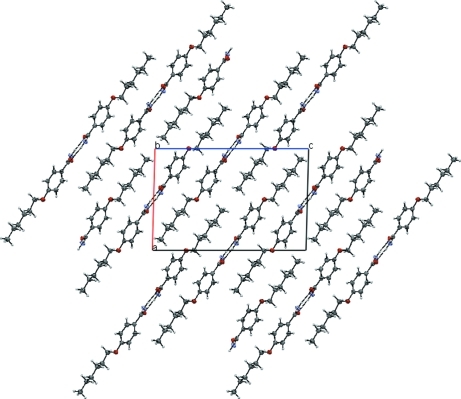
Packing diagram of the title compound viewed along the a axis; hydrogen bond are shown as dashed lines.

## supplementary crystallographic information

### Comment

Bond distance and angles in the titled compound (I), 4-(hexyloxy)benzamide (Fig.1) are in normal range (Allen *et al.* 1987) and are comparable with those in closely related structure which was determined by single-crystal X-ray diffraction method (Merz, 2002; Jones *et al.*, 2002). On the other hand, bond distances at ether and amide group differ from those in the structure of 2-ethoxybenzamide (Pagola & Stephens, 2009). The benzene ring with O1 and C8 is nearly planar (r.m.s deviation of 0.0004 Å), whereas C7 is 0.019Å out of this plane. The dihedral angle between the benzene ring and the amide group plane is 29.3° [20.1° in *p*-nitrobenzamide (Jones *et al.*, 2002)].In the molecular structure of (I) alkane carbon skeleton is not coplanar with the aromatic ring; the dihedral angle between the benzene ring and the alkane carbon skeleton is 5.40 (1)°. The torsion angles along the alkane carbon skeleton increase (torsion angle of C11—C14=174.1°, C12—C15=175.9°, and C13—C16=178.2°). The arrangement observed here is slightly different from n-alkanes which contain a planar zigzag carbon skeleton. However, the mean C(H3)—C(H2) and C(H2)—C(H2) distance, and C(H3)—C(H2)—C and C(H2)—C(H2)—C angles, are in agreement with those determined for n-alkanes [1.521 (1)Å and 112.8 (1)° and 113.5 (1)°, respectively; Boese *et al.*, 1999]. In the crystal structure, the amide groups are oriented head-to-head forming N10—H102···O9 hydrogen bond at (-*x*, -*y* + 1, -*z* + 1) [the N···O distance is 1.941 Å] to generate a hydrogen bond dimer. These dimers are further linked together by N10—H101···O9 hydrogen bonding at (*x*,*y* + 1, *z*) [N···O distance is 3.106 Å] generating a ladder-like motif along the *b* axis (Table 1, Figs. 2 and 3) (Pagola & Stephens, 2009).

### Experimental

Attempts to crystallize 1,2-bis(4-heptylbenzylidene)hydrazine by liquid diffusion method from n-buthanol and water led to crystals of the title compound, presumably due to slow hydrolysis by supervenient of water (Gallardo & Begnini, 1995; Pala Wilgus *et al.*, 1995).

### Refinement

The H atoms were all located in a difference map, but those attached to carbon atoms were repositioned geometrically. The H atoms were initially refined with soft restraints on the bond lengths and angles to regularize their geometry (C—H in the range 0.93–0.98, N—H in the range 0.86–0.90 Å) and *U*_iso_(H) (in the range 1.2–1.5 times *U*_eq_ of the parent atom), after which the positions were refined with riding constraints.

### Crystal data

**Table:**

C_13_H_19_NO_2_	*F*(000) = 480
*M**_r_* = 221.30	*D*_x_ = 1.198 Mg m^−^^3^
Monoclinic, *P*2_1_/*n*	Cu *K*α radiation, λ = 1.54184 Å
Hall symbol: -P 2yn	Cell parameters from 8720 reflections
*a* = 12.5507 (2) Å	θ = 3.5–70.8°
*b* = 5.16441 (9) Å	µ = 0.64 mm^−^^1^
*c* = 18.9322 (3) Å	*T* = 150 K
β = 91.4702 (16)°	Needle-like, colourless
*V* = 1226.72 (4) Å^3^	0.31 × 0.08 × 0.05 mm
*Z* = 4	

### Data collection

**Table:**

Oxford Diffraction Gemini E diffractometer	2355 independent reflections
Radiation source: sealed x-ray tube	2165 reflections with *I* > 2σ(*I*)
graphite	*R*_int_ = 0.018
ω/2θ scans	θ_max_ = 71.0°, θ_min_ = 4.2°
Absorption correction: multi-scan (*CrysAlis PRO*: Oxford Diffraction, 2006)	*h* = −15→13
*T*_min_ = 0.894, *T*_max_ = 1.000	*k* = −6→6
13258 measured reflections	*l* = −21→23

### Refinement

**Table:**

Refinement on *F*^2^	Primary atom site location: structure-invariant direct methods
Least-squares matrix: full	Hydrogen site location: inferred from neighbouring sites
*R*[*F*^2^ > 2σ(*F*^2^)] = 0.037	H-atom parameters constrained
*wR*(*F*^2^) = 0.095	Method = Modified Sheldrick *w* = 1/[σ^2^(*F*^2^) + (0.05*P*)^2^ + 0.41*P*], where *P* = [max(*F*_o_^2^,0) + 2*F*_c_^2^]/3
*S* = 1.01	(Δ/σ)_max_ = 0.0002608
2101 reflections	Δρ_max_ = 0.18 e Å^−^^3^
145 parameters	Δρ_min_ = −0.18 e Å^−^^3^
0 restraints	

### Special details

**Table:**

Refinement. For this compound, 13258 reflections were measured and collected during the refinement. However after merging the symmetry equivalent reflections, there were only 2355 independent reflections. Further 254 more reflections were filtered, as sigma cutoff was set at 3.0 and (sin theta x 2)set to >0.01 (to eliminate reflection measured near the vicinity of beam stop) therefore reduced the number of reflection to 2101 which were used in the Refinement.

### Fractional atomic coordinates and isotropic or equivalent isotropic displacement parameters (Å^2^)

**Table:**

	*x*	*y*	*z*	*U*_iso_*/*U*_eq_	
O1	0.50007 (7)	0.75339 (18)	0.27977 (5)	0.0339	
C2	0.40797 (9)	0.7236 (2)	0.31525 (6)	0.0276	
C3	0.40594 (10)	0.5166 (2)	0.36254 (7)	0.0327	
C4	0.31610 (10)	0.4686 (2)	0.40089 (6)	0.0302	
C5	0.22742 (9)	0.6304 (2)	0.39492 (6)	0.0258	
C6	0.23026 (10)	0.8374 (2)	0.34797 (6)	0.0283	
C7	0.31893 (10)	0.8824 (2)	0.30718 (6)	0.0292	
C8	0.13215 (9)	0.5718 (2)	0.43773 (6)	0.0265	
O9	0.11152 (7)	0.34604 (16)	0.45547 (5)	0.0322	
N10	0.07033 (9)	0.7708 (2)	0.45501 (6)	0.0326	
C11	0.50247 (10)	0.9496 (3)	0.22591 (7)	0.0334	
C12	0.61157 (10)	0.9498 (2)	0.19414 (7)	0.0337	
C13	0.64375 (10)	0.6928 (2)	0.16146 (7)	0.0321	
C14	0.74928 (10)	0.7091 (2)	0.12345 (7)	0.0314	
C15	0.78664 (10)	0.4500 (3)	0.09497 (7)	0.0339	
C16	0.89010 (11)	0.4699 (3)	0.05528 (7)	0.0392	
H31	0.4688	0.4087	0.3672	0.0424*	
H41	0.3139	0.3171	0.4318	0.0397*	
H61	0.1691	0.9533	0.3429	0.0368*	
H71	0.3177	1.0309	0.2744	0.0385*	
H112	0.4882	1.1252	0.2475	0.0433*	
H111	0.4461	0.9082	0.1885	0.0424*	
H122	0.6652	1.0029	0.2319	0.0438*	
H121	0.6111	1.0874	0.1576	0.0426*	
H131	0.6479	0.5555	0.1986	0.0402*	
H132	0.5859	0.6413	0.1263	0.0417*	
H141	0.8049	0.7782	0.1575	0.0417*	
H142	0.7433	0.8391	0.0847	0.0407*	
H151	0.7949	0.3232	0.1343	0.0435*	
H152	0.7299	0.3781	0.0628	0.0445*	
H162	0.9124	0.2983	0.0389	0.0627*	
H163	0.9472	0.5385	0.0870	0.0619*	
H161	0.8823	0.5875	0.0142	0.0630*	
H101	0.0938	0.9339	0.4498	0.0433*	
H102	0.0124	0.7437	0.4808	0.0432*	

### Atomic displacement parameters (Å^2^)

**Table:**

	*U*^11^	*U*^22^	*U*^33^	*U*^12^	*U*^13^	*U*^23^
O1	0.0292 (4)	0.0377 (5)	0.0350 (5)	0.0032 (4)	0.0051 (4)	0.0078 (4)
C2	0.0281 (6)	0.0268 (6)	0.0279 (6)	−0.0015 (5)	0.0011 (4)	−0.0021 (5)
C3	0.0319 (6)	0.0296 (6)	0.0365 (7)	0.0059 (5)	0.0000 (5)	0.0042 (5)
C4	0.0367 (7)	0.0238 (6)	0.0300 (6)	0.0010 (5)	0.0007 (5)	0.0046 (5)
C5	0.0312 (6)	0.0199 (5)	0.0264 (5)	−0.0024 (4)	0.0004 (4)	−0.0026 (4)
C6	0.0317 (6)	0.0209 (6)	0.0325 (6)	0.0024 (5)	0.0019 (5)	0.0006 (5)
C7	0.0346 (6)	0.0220 (6)	0.0311 (6)	−0.0002 (5)	0.0029 (5)	0.0039 (5)
C8	0.0331 (6)	0.0216 (6)	0.0248 (5)	−0.0019 (5)	0.0001 (5)	−0.0006 (4)
O9	0.0390 (5)	0.0201 (4)	0.0378 (5)	−0.0009 (3)	0.0095 (4)	0.0028 (3)
N10	0.0382 (6)	0.0204 (5)	0.0399 (6)	−0.0006 (4)	0.0136 (5)	0.0011 (4)
C11	0.0356 (7)	0.0278 (6)	0.0373 (7)	0.0011 (5)	0.0070 (5)	0.0042 (5)
C12	0.0353 (7)	0.0279 (6)	0.0382 (7)	−0.0037 (5)	0.0071 (5)	0.0001 (5)
C13	0.0316 (6)	0.0277 (6)	0.0370 (7)	−0.0031 (5)	0.0030 (5)	−0.0013 (5)
C14	0.0335 (6)	0.0271 (6)	0.0335 (6)	−0.0027 (5)	0.0024 (5)	−0.0002 (5)
C15	0.0335 (7)	0.0292 (7)	0.0391 (7)	−0.0018 (5)	0.0013 (5)	−0.0039 (5)
C16	0.0368 (7)	0.0393 (8)	0.0415 (7)	0.0014 (6)	0.0031 (6)	−0.0071 (6)

### Geometric parameters (Å, °)

**Table:**

O1—C2	1.3605 (14)	C11—H112	1.013
O1—C11	1.4384 (15)	C11—H111	1.011
C2—C3	1.3954 (17)	C12—C13	1.5233 (17)
C2—C7	1.3915 (17)	C12—H122	1.007
C3—C4	1.3792 (17)	C12—H121	0.992
C3—H31	0.968	C13—C14	1.5258 (17)
C4—C5	1.3943 (17)	C13—H131	0.998
C4—H41	0.978	C13—H132	1.007
C5—C6	1.3912 (16)	C14—C15	1.5216 (17)
C5—C8	1.4926 (16)	C14—H141	1.003
C6—C7	1.3904 (17)	C14—H142	0.996
C6—H61	0.976	C15—C16	1.5203 (18)
C7—H71	0.986	C15—H151	0.995
C8—O9	1.2423 (14)	C15—H152	0.997
C8—N10	1.3338 (15)	C16—H162	0.982
N10—H101	0.898	C16—H163	0.989
N10—H102	0.898	C16—H161	0.990
C11—C12	1.5096 (17)		
			
C2—O1—C11	117.58 (9)	C11—C12—C13	114.45 (10)
O1—C2—C3	115.66 (11)	C11—C12—H122	108.3
O1—C2—C7	124.71 (11)	C13—C12—H122	110.2
C3—C2—C7	119.63 (11)	C11—C12—H121	106.8
C2—C3—C4	120.28 (11)	C13—C12—H121	109.7
C2—C3—H31	118.1	H122—C12—H121	106.9
C4—C3—H31	121.6	C12—C13—C14	112.69 (10)
C3—C4—C5	120.75 (11)	C12—C13—H131	110.0
C3—C4—H41	119.7	C14—C13—H131	110.0
C5—C4—H41	119.5	C12—C13—H132	107.7
C4—C5—C6	118.62 (11)	C14—C13—H132	108.8
C4—C5—C8	118.89 (10)	H131—C13—H132	107.5
C6—C5—C8	122.47 (11)	C13—C14—C15	113.43 (10)
C5—C6—C7	121.16 (11)	C13—C14—H141	108.4
C5—C6—H61	120.1	C15—C14—H141	109.0
C7—C6—H61	118.8	C13—C14—H142	109.6
C2—C7—C6	119.50 (11)	C15—C14—H142	110.5
C2—C7—H71	121.8	H141—C14—H142	105.7
C6—C7—H71	118.6	C14—C15—C16	112.97 (11)
C5—C8—O9	120.85 (10)	C14—C15—H151	109.9
C5—C8—N10	117.12 (10)	C16—C15—H151	109.9
O9—C8—N10	122.03 (11)	C14—C15—H152	108.8
C8—N10—H101	120.1	C16—C15—H152	109.2
C8—N10—H102	119.9	H151—C15—H152	105.8
H101—N10—H102	118.5	C15—C16—H162	110.4
O1—C11—C12	108.58 (10)	C15—C16—H163	109.8
O1—C11—H112	109.7	H162—C16—H163	107.9
C12—C11—H112	109.4	C15—C16—H161	111.2
O1—C11—H111	108.7	H162—C16—H161	109.2
C12—C11—H111	110.3	H163—C16—H161	108.3
H112—C11—H111	110.1		

### Hydrogen-bond geometry (Å, °)

**Table:**

*D*—H···*A*	*D*—H	H···*A*	*D*···*A*	*D*—H···*A*
N10—H101···O9^i^	0.90	2.14	3.0153 (18)	164
N10—H102···O9^ii^	0.90	2.04	2.9401 (18)	174

Symmetry codes: (i) *x*, *y*+1, *z*; (ii) −*x*, −*y*+1, −*z*+1.

## References

[bb1] Allen, F. H., Kennard, O., Watson, D. G., Brammer, L., Prpen, A. G. & Taylor, R. (1987). *J. Chem. Soc. Perkin Trans. 2*, pp. S1–19.

[bb2] Altomare, A., Cascarano, G., Giacovazzo, C., Guagliardi, A., Burla, M. C., Polidori, G. & Camalli, M. (1994). *J. Appl. Cryst.* **27**, 435.

[bb3] Betteridge, P. W., Carruthers, J. R., Cooper, R. I., Prout, K. & Watkin, D. J. (2003). *J. Appl. Cryst.* **36**, 1487.

[bb4] Boese, R., Weiss, H.-C. & Blaeser, D. (1999). *Angew. Chem. Int. Ed.* **38**, 988–992. 10.1002/(SICI)1521-3773(19990401)38:7<988::AID-ANIE988>3.0.CO;2-0 29711877

[bb5] Gallardo, H. & Begnini, I. M. (1995). *Mol. Cryst. Liq. Cryst. Sci. Technol.* **258**, 85–94.

[bb6] Jones, P. G., Thönnessen, H., Schmutzler, R. & Fischer, A. K. (2002). *Acta Cryst.* E**58**, o1436–o1438.

[bb7] Macrae, C. F., Edgington, P. R., McCabe, P., Pidcock, E., Shields, G. P., Taylor, R., Towler, M. & van de Streek, J. (2006). *J. Appl. Cryst.* **39**, 453–457.

[bb8] Merz, K. (2002). *Acta Cryst* E**58**, o450–o451.

[bb9] Oxford Diffraction (2006). *CrysAlis CCD* and *CrysAlis RED* Oxford Diffraction Ltd, Abingdon, England.

[bb10] Pagola, S. & Stephens, P. W. (2009). *Acta Cryst.* C**65**, o583–o586. 10.1107/S0108270109040803 19893241

[bb11] Pala Wilgus, C., Downing, S., Molitor, E., Bains, S., Pagni, R. M. & Kabalka, G. W. (1995). *Tetrahedron Lett.* **36**, 3469–3472

